# Nodule–Microbiome Dynamics: Deciphering the Complexities of Nodule Symbiosis and the Root Microbiome

**DOI:** 10.3390/ijms27031487

**Published:** 2026-02-02

**Authors:** Raja Ben-Laouane, Mohamed Ait-El-Mokhtar, Abdelilah Meddich, Marouane Baslam

**Affiliations:** 1 FSTE-FSM Joint Laboratory “Natural Resources, Health, and Environment”-UMI, Bioresources, Environment and Health Research Team, Faculty of Science and Technology of Errachidia, Moulay Ismail University of Meknes, Marjane 2, BP 298, Meknes 50050, Morocco; 2 Center of Agrobiotechnology and Bioengineering, Research Unit Labelled CNRST (Centre AgroBiotech-URL-7 CNRST-05), Cadi Ayyad University, Marrakesh 40000, Morocco; a.meddich@uca.ma; 3 Laboratory of Biotechnology, Agri-Food, Materials, and Environment (LBAME), Department of Biology, Faculty of Science and Technology Mohammedia, Hassan II University of Casablanca, Mohammedia 28800, Morocco; mohamed.aitelmokhtar@gmail.com; 4 African Sustainable Agriculture Research Institute (ASARI), University Mohammed VI Polytechnic (UM6P), Laayoune 70000, Morocco; 5 GrowSmart, Paul van Vlissingenstraat 10 F, 1096BK Amsterdam, The Netherlands

**Keywords:** endophytic diversity, microbiome dynamics, nodule microbiota, rhizobial communication, signaling, symbiosis

## Abstract

Microbiomes play a pivotal role in sustaining plant function and broader ecosystem processes. Leguminous plants host vast populations of intracellular bacteria within specialized root organs known as nodules. The intricate mutualism between legumes and rhizobia ensures a stable supply of biologically fixed nitrogen (N) essential for plant growth. While rhizobia remain the central actors in this symbiosis, recent discoveries reveal the presence of non-rhizobial endophytes within nodules, suggesting a complex interplay shaped by host selection and compatibility with rhizobial partners. Understanding the structure and dynamics of crop nodule-associated microbial communities is thus critical for optimizing host responses to rhizobia and for leveraging beneficial plant–microbe interactions. This review explores the dualistic nature—both facilitative and inhibitory—of the nodule microbiome in relation to nodulation. We examine the diversity of soil bacteria that stimulate nodulation and those that ultimately colonize nodule tissues, questioning whether these functional groups overlap. Furthermore, we discuss the molecular dialogs and counter-signaling mechanisms that regulate endophyte ingress into nodules, and evaluate how nodule endophytes contribute to plant performance and soil fertility.

## 1. Introduction

Leguminous plants are of profound agronomic, ecological, and economic importance owing to their capacity to form mutualistic symbioses with *Rhizobium* species, enabling atmospheric nitrogen (N_2_) utilization [1,2]. This symbiosis underpins biological nitrogen fixation (BNF), which contributes approximately 11.1 million metric tons of fixed N annually in developing regions [3]. Beyond their direct productivity benefits, legumes enhance soil fertility, play an indispensable role in crop rotation, and reduce dependence on synthetic fertilizers [4,5,6]. Despite the atmospheric abundance of nitrogen, its bioavailability in soils often limits productivity, making diazotrophic bacteria—organisms equipped with the nitrogenase enzyme complex—vital for converting atmospheric N_2_ into plant-available forms [7,8,9].

In this mutualism, the host plant provides carbon substrates and energy to its bacterial symbionts, which in turn fix atmospheric nitrogen into ammonia for plant assimilation [10,11,12]. This ammonia is rapidly assimilated by the plant and subsequently incorporated into organic nitrogen compounds, reflecting a coevolved integration of carbon and nitrogen metabolism. Across legume species, this integration has driven diversification in nitrogen transport strategies, with fixed nitrogen being exported predominantly as amides in some legumes or converted into ureides in others, such as soybean [13,14]. This partnership exemplifies the ecological and biotechnological potential of plant–microbe alliances to enhance productivity while supporting environmental stewardship. The industrial synthesis of mineral nitrogen fertilizers remains highly energy-intensive [15], with N fertilizers accounting for up to 32% of the total operational energy costs in certain crops [16]. Globally, the combined economic and environmental burden of mineral fertilizer production exceeds USD 100 billion annually [16]. In this context, microbial inoculants based on N-fixing bacteria represent a sustainable alternative [17,18,19,20]. However, the success of such inoculants depends on their persistence and functional stability within the soil microbiome, which in turn is shaped by multiple biotic and abiotic factors [21,22]. A holistic understanding of plant microbiota dynamics, extending beyond the binary legume–rhizobium association, is thus indispensable.

The legume–rhizobium symbiosis remains one of the most efficient systems for BNF [23]. While ecologically secure, this interaction is restricted to specific plant groups—principally legumes forming nodules with *Rhizobium*, and actinorhizal species associating with filamentous *Frankia* bacteria to form actinorhizae. Non-symbiotic nitrogen fixation also occurs in non-leguminous plants that interact with free-living diazotrophs such as *Nitrospirillum amazonense*, *Gluconacetobacter diazotrophicus*, *Herbaspirillum seropedicae*, *Herbaspirillum rubrisubalbicans*, and *Burkholderia* spp. within the sugarcane rhizosphere [24]. Plant roots attract 2–10 times more bacterial taxa than leaves, and the root microbiome is dynamically regulated by soil physicochemical properties (pH, moisture, temperature) and plant factors such as genotype and developmental stage [25].

**Figure 1:**
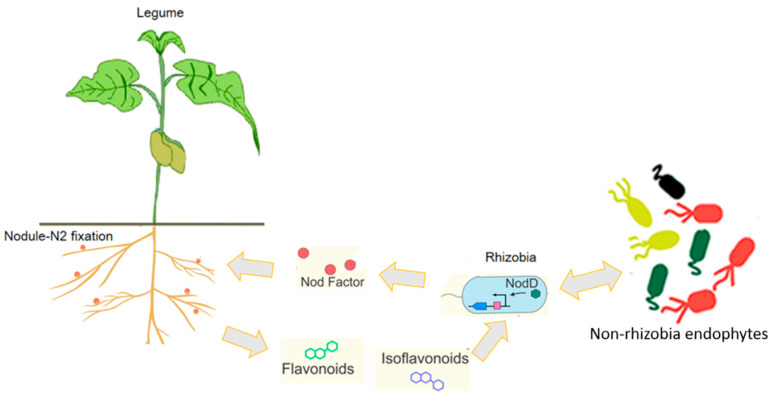
Inter- and intra-kingdom communication in the rhizosphere and signal exchange during the legume–rhizobium interaction.

Nodulation is initiated through specific molecular recognition between rhizobia and legume roots, mediated by reciprocal signaling [26,27,28]. Under ammonium-deficient conditions, root hairs exude phenolic compounds—predominantly flavonoids such as methoxychalcone—that act as chemo-attractants and signaling molecules. These flavonoids, particularly potent in *Medicago* and other legumes forming indeterminate nodules [29,30], induce bacterial *nod* gene expression, leading to the biosynthesis of lipochitooligosaccharide Nod factors. These molecules trigger plant signaling cascades that recognize compatible rhizobia and initiate cortical cell division to form nodules. Within nodules, bacteria differentiate into nitrogen-fixing bacteroids enclosed by the plant-derived peribacteroid membrane [31]. This tightly regulated process exemplifies the co-evolution of inter-kingdom communication systems ensuring infection control, bacteroid differentiation, and long-term symbiotic persistence [27,32,33] (Figure 1).

For decades, rhizobia were believed to be the exclusive occupants of legume nodules. However, recent metagenomic and culture-dependent studies have challenged this view, revealing diverse non-rhizobial microorganisms inhabiting nodules [34,35,36]. Legumes, like other plants, coexist with a multifaceted microbiota comprising bacteria, archaea, fungi, and protists. These communities can modulate nodulation outcomes—enhancing, neutralizing, or inhibiting symbiotic performance—and thus influence plant growth and productivity.

In this review, we dissect the composition and function of the nodule microbiome, addressing both its beneficial and adverse interactions with nodulation. We emphasize shared signaling processes underpinning progressive plant–microbe engagement, highlight the interplay of communication and counter-communication mechanisms that regulate endophyte access to host nodules, and examine the broader ecological implications of nodule-associated endophytes in promoting plant growth and soil fertility.

## 2. Deciphering the Nodule Microbiome: Composition of Nodule-Associated Microbiome and Structuring Factors

### 2.1. Unveiling the Microbial Tapestry—The Bacterial and Archaeal Microbiota of Nodule

**Figure 2:**
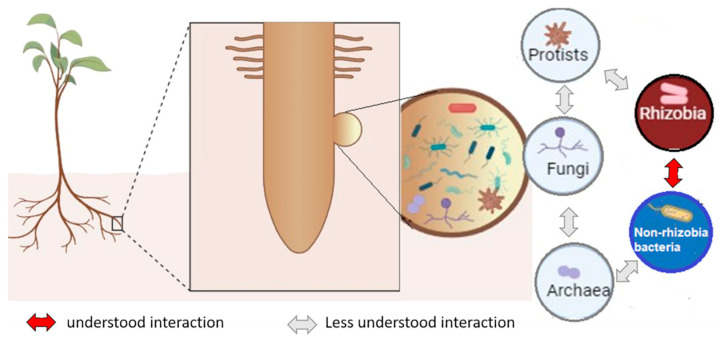
The complex microbial consortia naturally inhabiting the root nodules of leguminous plants.

The legume nodule represents a unique and dynamic microhabitat that accommodates an exceptionally diverse consortium of microorganisms. The principal constituents of this assemblage include rhizobia, non-rhizobial bacteria, archaea, fungi, and protists. Each component contributes distinct ecological and functional attributes that collectively influence nodulation efficiency, nitrogen fixation, and overall plant health (Figure 2). The intricate web of microbial interactions within nodules—mediated through signaling molecules, molecular exchanges, and inter-species communication—plays a decisive role in determining the outcome of the legume–microbe partnership. Disentangling the composition and ecological drivers of this consortium is therefore critical to understanding how nodule-associated microorganisms shape soil fertility, host productivity, and the resilience of leguminous crops.

Although numerous bacterial phyla coexist in nature and are subject to myriad environmental influences, the plant microbiome—both above- and below-ground—is typically dominated by members of Proteobacteria, Actinobacteria, and Bacteroidetes [37,38]. Host-associated microbiota exhibit organ-specific specialization and adaptation [39]. Within roots, the nodule microbiota encompasses not only nitrogen-fixing rhizobia but also a wide array of non-rhizobial bacteria. Genera such as *Pseudomonas* spp., *Klebsiella* spp., and *Rhodococcus* spp. have been repeatedly isolated from nodules [34]. In soybean nodules cultivated under saline and alkaline conditions, endosymbionts belonging to Actinobacteria, Bacteroidetes, Chloroflexi, Acidobacteria, and Gemmatimonadetes—including *Sphingomonas* spp., *Microbacterium* spp., *Arthrobacter* spp., *Nocardioides* spp., *Streptomyces* spp., *Flavobacterium* spp., *Flavisolibacter* spp., and *Pseudomonas* spp., along with members of the *Enterobacteriaceae*—have been identified [40].

In *Lotus parviflorus* Desf., only ≈ 10% of nodule isolates are genuine symbionts, while the remaining ≈ 90% represent opportunistic nodule-associated bacteria. The nodulating isolates belong predominantly to *Bradyrhizobium* spp., whereas non-nodulating taxa comprise α-proteobacteria (*Rhizobium* spp./*Agrobacterium* spp.), β-proteobacteria (*Massilia* spp.), and γ-proteobacteria (*Pseudomonas* spp., *Lysobacter* spp., *Luteibacter* spp., *Stenotrophomonas* spp., *Rahnella* spp.), in addition to bacteroid genera such as *Sphingobacterium* spp. and *Mucilaginibacter* spp. [41]. *Agrobacterium* species, often co-isolated with *Sinorhizobium meliloti* in *Melilotus dentatus* (Waldst. & Kit.) Desf. nodules, exemplify such cohabitation [42]. A recently described species, *Mesorhizobium onobrychidis*, isolated from *Onobrychis viciifolia* Scop. nodules, induces nodulation and fixes both N_2_ and CO_2_ despite lacking motility genes. Its genome harbors a distinctive chromosomal island enriched in symbiotic determinants and plant-growth-promotion genes, highlighting the adaptive plasticity of the genus [43].

Non-nodulating bacteria typically gain access to nodules through infection threads formed by compatible rhizobia and subsequently colonize internal tissues [44]. Although incapable of initiating nodules independently, these taxa can enhance nodulation when co-inoculated with rhizobia, exhibiting diverse plant-growth-promoting (PGP) traits [45]. Such mechanisms include increased phosphate and potassium solubilization and the production of siderophores, exopolysaccharides, and indole-3-acetic acid (IAA) [34]. In *Medicago truncatula* Gaertn, non-rhizobial inhabitants produce antimicrobial metabolites that modulate community structure and function [46]. Endophytic bacteria from *Sphaerophysa salsula*—notably *Mesorhizobium* spp. and *Bacillus* spp.—display IAA synthesis, ACC-deaminase activity, phosphate solubilization, chitinase, siderophore production, and antifungal potential, collectively enhancing nodulation and N fixation through synergism with rhizobia [47].

Beyond the existence of plant growth promoting rhizobacteria (PGPR) in the nodular microbiome, numerous experimental studies have examined the co-inoculation of legumes with nodular rhizobia (such as *Bradyrhizobium*, *Rhizobium*) and PGPR, particularly *Azospirillum* spp., *Bacillus* spp., *Pseudomonas* spp. or *Paenibacillus* spp. [48,49]. All of this research consistently demonstrates beneficial results on (i) root architecture and extent of soil exploration, (ii) the precocity via hormonal regulation and/or extent of nodulation, (iii) stress mitigation through ACC deaminase production and other mechanisms, (iv) nutrient availability (phosphorus solubilization, potassium mobilization, nitrogen assimilation) and, in some cases, yield, with the responses observed depending on the specific plant species, genotype, soil characteristics, and stress conditions [48,50]. These positive effects underscore that multiple functional categories of PGPR may act synergistically with rhizobia, although the outcome is strongly dependent on plant genotype soil characteristics, and stress conditions [48].

Microbial interactions within the nodule can shape symbiotic efficiency via antimicrobial competition [51], pathogen suppression [46,52,53], and horizontal gene transfer [34,54]. The assembly and persistence of these communities are influenced by a constellation of factors, including soil type, plant compartment, host genotype, developmental stage, nodulation pathway, immune responses, and seasonal or residence-time dynamics [39,55]. Host-driven selection strongly governs microbiota composition: *Lotus japonicus* (Regel) K. Larsen mutants defective in N-fixing symbiosis exhibit marked shifts in both root and rhizosphere communities, affecting ≥ 14 bacterial orders [55]. Compared with bacterial diversity, archaeal biology remains comparatively underexplored. Yet rapid advances in high-throughput sequencing, metagenomic assembly, and single-cell genomics have revolutionized archaeal systematics, enabling the delineation of ≥ 27 phyla [56,57]. A global phylogenomic survey of 3599 archaeal genomes based on conserved ribosomal markers has refined the archaeal tree of life into three superphyla: Asgard, DPANN, and TACK [56]. Despite limited study, archaea are increasingly recognized as integral members of plant microbiomes [58]. Endophytic archaeal lineages—principally *Thaumarchaeota*, *Crenarchaeota*, and *Euryarchaeota*—have been detected in roots and nodules, although their functional relevance to the host remains largely unresolved [52].

The *Thaumarchaeota* and *Crenarchaeota* belong to the TACK superphylum, while *Euryarchaeota* currently stand apart [56,59]. Given the modest size of the archaeal genomic dataset, taxonomic frameworks remain provisional and are likely to evolve as sampling expands [60]. Many archaea thrive under extreme environmental conditions, yet their detection within plant tissues suggests physiological versatility. Genomic and metagenomic analyses reveal archaeal genes involved in amino-acid biosynthesis, phytohormone modulation, and ammonia oxidation [61]. Ammonia-oxidizing archaea, in particular, may mitigate abiotic stress by regulating rhizospheric pH and nitrogen turnover [56,61]. Furthermore, archaeal endophytes exhibit classical PGP attributes—nitrogen fixation, phosphate solubilization, siderophore and IAA production, sulfur cycling, ammonia oxidation, and dissimilatory nitrate reduction [62,63,64,65]. These features position archaea as promising agents for sustainable agriculture under climate-change pressures.

Despite their reputation as extremophiles, archaea exert tangible ecological functions in terrestrial ecosystems through interactions with both biotic and abiotic factors [56]. Within holobiont systems, *Euryarchaeota* (methanogenic, halophilic) and *Thaumarchaeota* species often coexist with bacteria and fungi, adapting to energy-limited niches via synergistic metabolism [66]. While prevalent in the rhizosphere and endosphere, archaeal abundance in the phyllosphere remains low, likely constrained by environmental stressors [67]. The diversity, ecology, and inter-kingdom interactions of archaea within nodules remain largely uncharacterized, although they are consistently detected in healthy plant tissues worldwide [67,68,69]. Recent methodological innovations offer new avenues for cultivating previously uncultured archaea. These include refining selective media based on genomic and transcriptomic cues, employing co-culture or direct inter-species electron-transfer systems, leveraging single-cell isolation and high-throughput microculture, and simulating natural microhabitats [70]. Nonetheless, archaeal cultivation remains hindered by unknown nutrient requirements and highly variable physicochemical preferences even among close relatives [71]. Continued innovation in culturing and in situ simulation technologies is therefore essential to elucidate archaeal physiology, ecological roles, and potential contributions to nodule assembly and function [66,72].

### 2.2. Unraveling the Lesser-Known Players of Nodules—The Fungal Microbiota

Although less studied than bacteria, the fungal microbiota exerts a crucial and dynamic influence on nodule microenvironments and plant–microbe interactions. The remarkable diversity of fungi colonizing aerial and root tissues—predominantly within Ascomycota and Basidiomycota—underscores the complexity of nodule-associated communities [39,53]. Within roots, arbuscular mycorrhizal fungi (AMF; Glomeromycota) and ectomycorrhizal taxa are well recognized; recent evidence further indicates that non-mycorrhizal endophytic fungi constitute a substantial fraction of the root mycobiome [39,73]. By contrast, the fungal constituents isolated specifically from plant nodules have received limited attention. Notably, interactions between nodule bacterial communities and fungi have been documented. For example, *Cupriavidus* spp., *Burkholderia* spp., and *Rhizobium* spp. were evenly represented on *Mimosa pudica* L. (38%, 37%, and 25% of isolates, respectively) [74], and *Burkholderia* spp. can associate with particular AMF lineages (e.g., *Gigaspora* spp., *Scutellospora* spp.), with up to ~250,000 bacteria per spore, highlighting the intrinsic capacity of mycorrhizae to harbor symbiotic bacteria [75].

Fungal endophytes form a major component of endophytic consortia. Most reported taxa belong to Ascomycota, Basidiomycota, and Mucoromycota [76,77]. Based on taxonomy, host range, transmission, and plant-health outcomes, endophytes are classed into two groups and four classes: Clavicipitaceous (Class I) and non-Clavicipitaceous (Classes II–IV) [78]. Clavicipitaceous endophytes are phylogenetically cohesive, vertically transmitted via seed, and restricted largely to cool- and warm-season grasses. In contrast, non-clavicipitaceous endophytes—mostly Ascomycota—are polyphyletic, occur across vascular and non-vascular plants, and often transition between endophytic and free-living lifestyles. They are subdivided by colonization patterns, intergenerational transmission, in planta diversity, and ecological function [77,79,80]. Class II taxa transmit both horizontally and vertically and colonize roots, shoots, and rhizomes; class III and IV transmit horizontally but colonize shoots and roots, respectively [78,81,82]. While endophytic fungal diversity has been cataloged across leaves, roots, seeds, and shoots [79], the nodule mycobiome remains largely unexplored. To our knowledge, the nodule mycobiome (plant–rhizobia–fungus symbiosis) was first characterized in *Acacia longifolia* (Andrews) Willd by Jesus et al. [82], although fungal communities had been described previously in the rhizosphere [68,83]. Reported fungal genera belonged to the phyla Ascomycota, Basidiomycota, and Mucoromycota, with *Coniochaeta* spp. dominant, followed by *Tubaria* spp., *Umbelopsis* spp., *Alternaria* spp., *Coprinellus* spp., *Tuber* spp., *Sclerotiophoma* spp., *Stromatinia* spp., *Dothiorella* spp., and *Thermothielavioides* spp. [82].

The nodule cortex likely provides a favorable microhabitat for fungal colonization. Fungi can benefit from rhizobial N fixation while supplying phosphorus to other partners, potentially enhancing the efficiency of the N-fixing symbiosis [84]. Ascomycota are frequently implicated in nutrient cycling, plant defense, and inter-organismal interactions [85,86]. Consistent with this, nodule-associated fungal endophytes may bolster nutrient acquisition and tolerance to abiotic and biotic stress, thereby improving plant growth and development. Nonetheless, many aspects of fungi–rhizobia interactions and the specific roles of fungal endophytes during nodule establishment remain unresolved and merit comprehensive investigation using integrative, multi-omic, and experimental approaches.

### 2.3. Unveiling the Enigmatic Players—Nodule-Associated Protists

Protists are among the least characterized constituents of soil, rhizosphere, and nodule microbiomes despite their ecological significance. Molecular surveys have assessed >10% of species within plant-associated protist communities; most known plant-interactive protists fall within the Stramenopiles–Alveolata–Rhizaria supergroup, particularly Oomycota (Stramenopiles) and Cercozoa (Rhizaria) [87,88]. Members of *Pythium*, *Phytophthora*, *Peronospora* (and related downy mildews), and *Albugo* are frequently associated with roots or leaves [39]. Protist community structure is shaped by multiple drivers, including seasonality; pronounced spatiotemporal heterogeneity has been observed in phytotelmata, with reduced heterogeneity during spring growth at a given site [89]. Although protists within nodules remain poorly documented, accumulating evidence suggests they influence nodule ecology and plant–microbe dynamics, underscoring the need for focused studies on diversity, abundance, and interactions across soil, plant tissues, and nodules [87]. Soil protists can actively redistribute beneficial bacteria along *Medicago truncatula* Gaertn roots [90]. Moreover, protist communities are reportedly more sensitive to N fertilization than other microbial groups in diverse agricultural soils [91]. Plant genotype-specific archaeal and bacterial endophytes—but similar *Bacillus antagonists*—colonize Mediterranean olive trees [92].

Across ecosystems, protists are pivotal to plant health, productivity, and soil fertility through nutrient cycling, selective grazing that restructures bacterial communities, and consumption of plant pathogens [90]. Functioning as top-down controllers of microbiomes linked to plant health, protists stimulate bacterial genes required for plant growth and suppress pathogens [93]. They also act as vectors for bacterial dispersal, including *Rhizobium*. For example, *Colpoda* sp. transports *Sinorhizobium meliloti* along *Medicago truncatula* Gaertn roots to sparsely populated microhabitats, facilitating deeper colonization and nodule formation [90]. Analogous “hitchhiking” phenomena occur elsewhere, such as fungal spores leveraging bacterial flagella [94].

The nodule cortex may constitute a suitable niche for protist colonization, with consequent effects on microbial efficiency within nodules and on host hormonal balance via shifts in microbial abundance and activity. Protists can enrich for IAA-producing bacteria, stimulating lateral root branching and increasing plant–microbe contact surfaces for nodulation [93]. They also elevate cytokinin levels, potentially through increased nitrate concentrations arising from excess N secretion [93]. Indirect hormonal modulation can occur through protist-driven changes in microbiome function—for instance, altered community-level production of 2,4-diacetylphloroglucinol (DAPG), an antimicrobial bacterial metabolite that interferes with auxin signaling [95]. Collectively, these hormonal and ecological effects likely contribute to the significant influence of protists on the nodule niche. As predators of plant-associated bacteria and fungi, dispersal agents, and modulators of community function, protists may be integral to nodule microbiome assembly and performance. Expanding protistology in plant systems will be essential to resolve their roles in structuring and sustaining nodule function.

### 2.4. Biological/Ecological Role of Rhizobial Volatile Compounds

Volatile organic compounds (VOCs) are small, low-molecular-weight molecules (<300 Da) encompassing a wide range of chemical classes and capable of diffusing readily through air- and water-filled pores [96]. These compounds play pivotal roles in promoting plant growth and enhancing resilience against both abiotic and biotic stresses. Numerous studies have identified plant growth promotion as a shared feature of volatile blends emitted by rhizosphere-associated bacteria [97,98,99,100,101,102]. VOCs can stimulate photosynthetic activity, root elongation, and nutrient assimilation in plants [103,104], although the molecular mechanisms underpinning these responses remain only partially elucidated.

From an ecological perspective, VOCs serve as chemical mediators within complex networks of plant, bacterial, fungal, and nematode interactions, influencing both intra- and interspecific microbial behavior [99,105,106]. They also contribute to stress tolerance by enhancing osmoprotectant accumulation and antioxidant defenses under adverse conditions [107,108]. Many VOCs exhibit dual functions—directly inhibiting pathogen growth and virulence while simultaneously activating plant immune responses [105]. Intriguingly, volatile blends released by rhizobacteria can reinforce mutualistic plant–microbe associations without compromising pathogen resistance, in a process modulated by plant phosphorus availability [109]. Depending on context, VOCs can exert either stimulatory or inhibitory effects on microbial communities: some display strong antibacterial or antifungal activity, while others act as signaling molecules capable of modulating gene expression at a distance [105]. Yet, the precise molecular mechanisms by which microorganisms perceive and respond to these volatiles—and how such interactions influence plant health and microbiome composition—remain largely unresolved.

Rhizobial volatile compounds (RVCs) represent a diverse subset of bacterial volatiles produced during rhizobial metabolism, encompassing numerous biologically active molecules (Table 1) [105]. These include fatty-acid derivatives such as hydrocarbons, ketones, and alcohols, alongside organic acids, sulfur- and nitrogen-containing volatiles, and terpenes [103]. Rhizobia and other soil bacteria also emit inorganic volatile compounds (VICs)—notably ammonia, nitric oxide (NO), hydrogen sulfide (H_2_S), and hydrogen cyanide (HCN)—which act as additional modulators of microbial and plant physiology. Both VOCs and VICs, spanning multiple chemical subclasses, have been extensively reviewed [96,98,103,106,110,111].

Although rhizobia are best known for their symbiotic nitrogen-fixing interactions with legumes, their volatile emissions constitute an underexplored dimension of interkingdom communication. RVCs are emerging as critical signaling agents influencing plant growth, microbial behavior, and community assembly. They have been shown to enhance plant vigor, bolster tolerance to environmental stress, and modulate induced systemic resistance (ISR) pathways, offering a promising avenue for improving crop resilience and sustainability [105]. Consequently, decoding the role of RVCs provides key insight into the chemical language mediating plant–microbe symbioses and their broader ecological implications. Compelling evidence indicates that plants can perceive and respond to volatile blends emitted by rhizobia [112]. RVC-mediated bioactivities include the stimulation of root development, activation of iron-uptake mechanisms, and upregulation of plant defense gene transcription [105]. The application of RVCs has been shown to influence both beneficial and pathogenic microorganisms and to impact leguminous as well as non-leguminous hosts. For example, bacterial volatiles can foster mutualistic interactions with beneficial rhizobacteria while maintaining disease resistance [104]. In both legume and non-legume systems, exposure to RVCs induces root iron-uptake pathways, a phenomenon potentially linked to defense activation, as observed for volatiles of other plant-beneficial rhizobacteria [113].

**Table 1:** Identified Rhizobial Volatile Compounds: Sources and Biological/Ecological Roles.

Rhizobial Volatile Compounds	Sources	Main Roles	Ref.
2-methyl-1-propanol and dimethyl-disulfide	*Sinorhizobium meliloti*	Activation of iron-uptake mechanisms, namely rhizosphere acidification and increased root ferric reductase in *Medicago truncatula* Gaertn	[114]
1,4-butanediol, 1,2,3-propanetriol monoacetate, triacetin, dehydroacetic acid, dimethyl phthalate, 3,5-diacetyl-2,6-dimethyl-4H-pyran-4-one	*Arthrobacter* *agilis* UMCV2, *Bacillus methylotrophicus* M4-96, *Sinorhizobium meliloti* 1021	Increase in chlorophyll content and transcriptional activity of iron-uptake genes in *Sorghum bicolor* (L.) Moench	[115]
Glyoxylic acid, 3-methyl-butanoic acid, diethyl acetic acid	*Bacillus subtilis* Cohn. GB03	Activation of Arabidopsis own iron acquisition machinery	[116]
N,N-dimethylhexadecylamine	*Arthrobacter agilis* UMCV2, *Sinorhizobium meliloti* 1021, or *Pseudomonas fluorescens*.	Promotion of plant growth and induction of iron-deficiency and defense response genes in *Medicago truncatula* Gaertn	[109]
Methylketone 2-tridecanone	*Sinorhizobium meliloti*	Increase in surface motility and defects in biofilm formation responsible for the pleiotropic phenotype of *Sinorhizobium meliloti* (Dangeard).	[117]
Albuterol and 1,3-propanediol	*Bacillus subtilis* SYST2	Promotion of tomato plant growth and differential expression of genes involved in auxin, gibberellin, cytokinin, expansin, and ethylene biosynthesis or metabolism	[104]
2R, 3R-butanediol, C13 VCs	*Bacillus subtilis* GB03, *Paenibacillus polymyxa*	Activation of plant defense responses	[113]
Phazolicin	*Rhizobium* spp. Pop5	Antimicrobial activity	[118]

RVCs display diverse and context-dependent effects on plant–microbe interactions. The antibiotic phazolicin, isolated from a *Rhizobium* sp. inhabiting *Phaseolus vulgaris* nodules, exhibits potent antimicrobial activity [118]. Similarly, RVCs produced by *Micromonospora* spp. and *Paenibacillus* spp. isolated from *Medicago sativa* L. nodules show pronounced antifungal activity against major phytopathogens under in vitro conditions [46,119]. Yet, fundamental questions persist regarding the precise influence of RVCs on host physiology and the establishment of effective symbiosis. It remains to be determined whether RVCs facilitate legume–rhizobium compatibility by inducing specific host signaling cascades that attract microsymbionts or prime the nodulation signaling pathway.

Overall, elucidating the ecological and functional roles of RVCs is essential for understanding the chemical basis of plant–microbe dialog. Such knowledge will not only deepen our grasp of nodule symbiosis and microbiome assembly but also inform the design of sustainable agricultural strategies leveraging microbial volatiles to enhance crop health and productivity.

The diversity of the players inside the nodule structure implies close and complicated interactions which will finetune the establishment of this ecosystem and its function.

## 3. Unraveling the Intricate Dance of Microbial Partners—The Dynamic and Complex Nature of Microbial Interactions Within the Nodule: Harmony and Rivalry

The root nodule microbiome constitutes a highly dynamic and adaptive ecosystem that evolves across plant developmental stages, harboring a multitude of microorganisms engaged in intricate webs of cooperation and competition [46,120]. Beyond their classical role as sites of biological nitrogen fixation, nodules function as metabolically active microenvironments enriched in specialized metabolites with ecological and physiological relevance [46]. Yet, the precise nature of microbial interactions and the in planta functions of these metabolites remain incompletely understood. Key aspects shaping the nodule ecosystem include (i) the balance between cooperative and antagonistic interactions among microbial inhabitants, (ii) the biosynthesis of specialized metabolites that mediate microbial coexistence, and (iii) the regulatory feedback between these metabolites and the broader plant–microbe interface. A mechanistic understanding of these processes is essential to decipher how nodule microbial consortia influence host productivity, nutrient acquisition, and stress resilience.

Microbial partners within the nodule—collectively referred to as nodule microbiome members (NMMs)—form complex interaction networks that fluctuate with environmental conditions and host developmental cues. These networks involve competitive, mutualistic, and syntrophic exchanges that shape nutrient fluxes, niche partitioning, and symbiotic efficiency [46]. NMMs may influence one another directly by transporting or transforming soil-derived substrates, modifying root exudation patterns, fixing nitrogen, secreting phytohormones, and producing signaling or inhibitory compounds that modulate partner behavior. Additionally, many bacteria synthesize exopolysaccharides, which confer protection against oxidative stress and aid in biofilm formation—an essential adaptation for survival within the nodule microenvironment [121]. The assembly and succession of nodule microbial communities are largely guided by plant-derived metabolites that act as chemoattractants, nutrient sources, or signaling molecules [122]. These plant–microbe–microbe feedback loops can result in positive, neutral, or antagonistic outcomes, depending on the compatibility and metabolic interdependence of the participating taxa. For instance, *Paenibacillus* sp. Ag47 and *Pseudomonas* sp. Ag54 exhibit a cooperative relationship during the early stages of nodule colonization, mutually enhancing colonization success. However, as the symbiosis matures, competitive exclusion dynamics emerge, reducing the persistence of *Paenibacillus* sp. Ag47 [46]. Similarly, *Pseudomonas* sp. Ag54 fails to establish nodular colonization when introduced alone but thrives in the presence of other community members, suggesting the existence of metabolic interdependencies or cross-feeding interactions that facilitate niche establishment.

Such examples underscore that nodule microbiomes are not static consortia but interactive metabolic networks, whose emergent properties—cooperation, competition, and communication—govern the stability and functionality of the symbiosis. The interplay between microbial community composition, metabolite exchange, and host physiology thus defines the delicate balance of harmony and rivalry that sustains the nodule ecosystem. Elucidating these dynamic interactions through multi-omic and ecological modeling approaches will be pivotal to understanding how nodule microbiomes co-evolve with their legume hosts to optimize nutrient efficiency and stress adaptation.

### 3.1. Orchestrating Harmony in Nodule Microbiomes: Cooperative Interactions Among Nodule Microbiome Members

#### 3.1.1. Nutrient Symphony: Cooperative Nutritional Interdependencies Among Nodule Microbiome Members

Within the nodule ecosystem, microbial partners engage in highly coordinated metabolic exchanges that drive nutrient cycling and sustain plant productivity. Through a suite of biochemical transformations, nodule-associated microorganisms mineralize organic forms of essential elements—N, P, S—into bioavailable inorganic compounds such as ammonium, nitrate, phosphate, and sulfate, which are fundamental to plant growth [4,123]. These cooperative interactions not only sustain the nutritional equilibrium of the nodule environment but also underpin broader ecosystem functions. Among these, phosphate solubilization and mobilization represent central processes in which diverse bacterial taxa contribute synergistically to enhancing nutrient availability, rendering them valuable agents for biofertilization.

The process of BNF—the enzymatic conversion of atmospheric N_2_ to ammonium—is energetically demanding and therefore tightly regulated. Its efficiency is strongly influenced by the availability of phosphorus, which functions as a key regulatory element. Quantitative proteomic analysis of *Azotobacter chroococcum* has revealed intricate links between BNF and phosphorus metabolism: N fixation induced changes in P-related pathways, including the upregulation of two key phosphatases—an exopolyphosphatase and a non-specific alkaline phosphatase (PhoX)—that drive P mobilization [124]. These findings further indicate that BNF can modulate the synthesis of nitrogenous bases and amino acids such as L-methionine, highlighting a previously unrecognized metabolic interdependence between N and P cycles [124]. Phosphate-solubilizing bacteria (PSB) enhance plant P nutrition by secreting organic acids and phosphatases that convert insoluble forms of P into plant-available forms [125]. Transcriptomic studies have demonstrated that genes encoding hydrolytic enzymes such as 1,3-β-glucanase and chitinase are co-regulated with IAA synthesis and P solubilization in *B. subtilis* and *Enterobacter* spp., revealing an integrated response that balances nutrient mobilization with growth-promoting activities [126].

Cooperative nutrient interactions are not limited to bacterial consortia but extend to AMF and rhizobial symbionts, which together form an integrated nutrient acquisition network. In the rice–AMF symbiosis, Wang et al. [127] identified the upregulation of nitrate transporter and assimilation genes—specifically members of the *NRT1*/*NPF* and *NRT2* families, including *OsNPF4.5*, *OsAMT*, and *OsAMT3.1*—within arbuscule-containing cortical cells. Notably, *OsNPF4.5* orthologs are expressed in AMF-colonized roots of diverse non-leguminous hosts such as maize, sorghum, and tomato, whereas the *Medicago ortholog* (*MtNPF4.5*) exhibits only slight induction in mycorrhizal roots [128]. Given that nitrate acts as a nodulation-suppressive signal in legumes, this weak induction in *Medicago* may represent a cooperative regulatory mechanism between AMF and rhizobia, maintaining a functional balance between mycorrhizal N uptake and symbiotic N fixation [129].

A multitude of P-solubilizing and mineralizing bacteria contribute to this cooperative nutrient economy. Genera such as *Azotobacter* spp., *Microbacterium* spp., *Bacillus* spp., *Burkholderia* spp., *Enterobacter* spp., *Flavobacterium* spp., *Erwinia* spp., *Rhizobium* spp., and *Serratia* spp. actively solubilize inorganic P and mineralize organic P, providing critical support for both plant growth and rhizobial activity [19,20,130,131]. These PSB function as natural biofertilizers, improving plant productivity through the synthesis of organic acids that chelate metal cations and release phosphate ions from otherwise insoluble mineral matrices [132].

AMF—including both arbuscular and vesicular–arbuscular mycorrhizal taxa—form extensive endosymbiotic networks that enhance nutrient acquisition and translocation, particularly for phosphorus and nitrogen [133,134]. Their hyphal networks create a shared mycelial matrix that interconnects plant roots, facilitating nutrient exchange and improving tolerance to environmental stresses [135]. AMF symbiosis has been shown to substantially enhance nitrogen fixation efficiency under P-limited conditions, primarily due to improved P uptake in mycorrhized plants compared to non-mycorrhized controls [136].

Molecular analyses of mycorrhiza-induced Pi transporters across multiple plant species have identified two conserved cis-regulatory motifs in their promoters: the P1BS element—common among P-starvation-induced genes—and MYCS (CTTC motif), a binding site for mycorrhiza-associated transcription factors [137]. Functional assays confirmed that both elements are essential for transcriptional activation of mycorrhiza-responsive P transporters. Regulation of these genes reflects an integrated control system responding simultaneously to plant P status and fungal colonization signals. Although the transcriptional regulators controlling these pathways remain incompletely defined, indirect evidence implicates RAM1 as a potential activator of symbiotic Pi transporters, with its effects varying among plant species [138].

Together, these findings illustrate that nutrient acquisition within nodules is not the product of isolated microbial activity but a symphonic interplay among bacterial, fungal, and plant partners. Cooperative interactions—linking nitrogen fixation, phosphorus mobilization, and symbiotic signaling—form the foundation of a metabolically interdependent network that sustains plant growth and contributes to ecosystem resilience. Understanding these nutrient-driven symbioses provides a powerful framework for developing next-generation biofertilization strategies to enhance sustainable agricultural productivity.

#### 3.1.2. Microbial Production of Various Regulators in Nodule Microbiomes

Microbial inhabitants of the nodule ecosystem are prolific producers of bioactive molecules that profoundly influence the physical, chemical, and developmental dynamics of plant roots. Among these compounds are signaling enzymes and phytohormones, notably the IAA—a central regulator of plant–microbe communication [139,140]. IAA biosynthesis by rhizospheric and endophytic bacteria has been demonstrated in a wide array of crops, with more than 80% of rhizosphere-associated isolates reported to produce auxins [141,142]. IAA plays a fundamental role in stimulating plant cell division, seed and tuber germination, adventitious root formation, and vascular differentiation, thereby modulating plant architecture and facilitating microbial colonization.

IAA production is widespread among diverse bacterial genera, including *Aeromonas* spp., *Azotobacter* spp., *Bacillus* spp., *Bradyrhizobium* spp., *Burkholderia* spp., *Enterobacter* spp., *Mesorhizobium* spp., *Pseudomonas* spp., *Rhizobium* spp., *Sinorhizobium* spp., Azospirillum and *Klebsiella* [143,144]. Many strains can synthesize IAA through multiple biosynthetic routes, which may operate either independently or dependently on tryptophan, the principal IAA precursor [145,146]. Beyond IAA, PGPR produce a suite of structurally related auxinic and aromatic compounds—such as indole lactic acid, indole-3-butyric acid, indole-3-propionic acid, indole-3-pyruvic acid, and tryptophol—that collectively contribute to the fine-tuning of plant developmental processes [14,147,148].

Cytokinins, another major class of phytohormones, are likewise synthesized by PGPR including *Arthrobacter* spp., *Bacillus* spp., *Azospirillum* spp., and *Pseudomonas* spp., [149,150]. These molecules positively influence root system architecture, cell division, and shoot development, thereby optimizing plant nutrient uptake [151]. The combined secretion of auxins and cytokinins by rhizobacteria enhances cross-kingdom interactions, particularly with AMF, by stimulating root elongation, lateral branching, and the expansion of root surface area [152,153].

Within this framework, *Rhizobium* spp. are especially prolific IAA producers, directly influencing vascular bundle formation and cortical cell expansion during early stages of nodulation. Phytohormone production by nodule-associated bacteria and fungi therefore not only modulates host physiology but also facilitates microbial networking within the root niche. Auxin- and cytokinin-mediated root remodeling increases potential colonization sites for both beneficial microbes and AMF, amplifying the likelihood of successful symbiotic establishment. Collectively, these regulatory molecules form a hormonal bridge linking plant development to microbiome assembly, underscoring the biochemical sophistication of the nodule ecosystem.

#### 3.1.3. Symbiotic Overture: Signaling Dynamics in Presymbiotic Stimulation in Nodule Microbiomes

The accommodation of symbionts and endophytes within nodules is orchestrated by the genetic and physiological machinery of the host plant, which governs microbial access and compatibility [44]. When leguminous plants encounter complex microbial communities, they exercise selective control over nodule colonization, thereby shaping the internal microbiome architecture. During the initiation of symbiosis, both microbial partners and host plants release a diverse spectrum of signal molecules, including phytohormones, enzymes, polysaccharides, phenolic compounds, adhesins, and volatile compounds, which coordinate the sequential stages of recognition, adhesion, and colonization [105,154].

In the mycorrhizosphere, bacteria can produce signaling metabolites that stimulate the presymbiotic growth of fungi and modulate mycorrhizal establishment. Conversely, bacterial degradation or transformation of fungal or plant-derived signal molecules can alter root–fungus recognition, influencing the outcome of the AMF–plant dialog [155]. For instance, bacterial culture filtrates have been shown to promote the hyphal extension of mycorrhizal fungi even on nutrient-poor media [156]. Bacteria may also detoxify inhibitory metabolites produced by fungi, thereby promoting mycelial expansion [157].

Rämä and Quandt [158], demonstrated that bacterial isolates obtained from sporocarps and spores of *Hebeloma crustuliniforme* and from *Salix* roots harboring the same mycorrhizal partner could induce AMF spore germination in co-culture. Germination was also enhanced by a co-occurring rust fungus, *Tritirachium roseum*, suggesting that multiple microbial partners can synergistically influence fungal developmental transitions. Such evidence underscores the importance of cross-kingdom signaling networks in shaping presymbiotic behavior and successful colonization. However, mechanistic understanding of bacterial–fungal interactions promoting AMF germination and establishment remains fragmentary.

Within the legume nodule context, the coordination of microbial infection is likewise governed by host genetic factors. In *Lotus japonicus* (Regel) K. Larsen, colonization of roots by *Mesorhizobium loti* facilitates the subsequent entry of other endophytic bacteria into nodules, where they attach to infection threads alongside nitrogen-fixing symbionts [44]. Metagenomic profiling further reveals that the bacterial communities inhabiting rhizosphere, root, and nodule compartments are assembled in parallel rather than sequentially [55]. This parallel recruitment process suggests a host-regulated gatekeeping mechanism in which legume plants selectively permit compatible microbes to cohabit the nodule while excluding others.

Altogether, these findings highlight that presymbiotic signaling is not confined to binary plant–microbe exchanges but involves an intricate choreography of bacterial, fungal, and host-derived signals. The legume host emerges as the principal conductor, modulating this symbiotic overture to ensure balanced cooperation between primary symbionts and auxiliary microbiome members. Understanding the molecular grammar of these early signaling dynamics will be pivotal to unraveling how nodules achieve such precise orchestration of microbial diversity and function.

In this context, in peas, transcriptomic analysis of *sym33* (*ipd3*/*cyclops*) mutants has identified several candidate regulators potentially involved in nodule differentiation, including CCS52, EFD, SYMREM, RSD, as well as members of the MADS-domain/AGL and SHORT INTERNODE/STYLISH transcription factor families. The regulation of the expression of these genes by cytokinin, suggested by their response to hormonal treatment, supports the hypothesis of the existence of a cytokinin-sensitive genetic network operating during the late stages of nodule development [159].

Complementarily, the study of *rms* mutants indicates that strigolactones also participate in the regulation of nodule development and maturation. The nodules of these mutants show accelerated development, associated with induction of nodule inception (NIN) and adjustments in carbon metabolism, suggesting that strigolactones, possibly in interaction with cytokinin, contribute to modulating the balance between nodule growth, nitrogen fixation, and senescence [160].

### 3.2. Engaging Rivalries Within Nodule Microbiomes

#### 3.2.1. Microbial Battlefront: Contact-Dependent Competitions in Nodule Microbiomes

Root nodules represent specialized ecological microhabitats that provide rhizobia with refuge from the intense microbial competition prevailing in bulk soil. Yet, this protection is not absolute. Mounting evidence reveals that nodules harbor a variety of non-rhizobial bacteria, indicating that rhizobia coexist—and at times directly compete—with other microbial residents within this confined niche. Despite increasing recognition of such mixed communities, the molecular and ecological mechanisms governing contact-dependent microbial competition inside nodules remain poorly understood.

Spatial organization within nodules likely reflects competitive exclusion among nodule microbiome members (NMMs), as spatially structured growth constrains resource overlap and enforces niche differentiation [161]. Hansen et al. demonstrated that previously cooperative interactions among nodule-associated bacteria can be destabilized by competitive pressures, leading to shifts in both community structure and symbiotic function [46]. Each NMM may exert antagonistic effects on rhizobia, implying that inter-bacterial competition can directly influence symbiotic efficiency and total nitrogen fixation in planta. Elucidating these antagonisms is therefore crucial for understanding how non-rhizobial residents modulate the legume–rhizobium partnership.

Field-based microbiome analyses have provided compelling empirical support for such dynamics. In field-grown peanut (*Arachis hypogaea* L.) inoculated with a commercial rhizobial strain, 16S rRNA sequencing revealed distinct microbial assemblages in nodules of different sizes [162]. Large nodules were overwhelmingly dominated by *Bradyrhizobium* (>99%) throughout the life cycle, whereas small nodules exhibited greater taxonomic diversity (~3%) comprising taxa absent from large nodules. Remarkably, these minor bacterial groups progressively increased in abundance in small nodules during late growth stages, suggesting competitive replacement between native soil bacteria and the commercial inoculant. Conversely, such competition was negligible in large nodules, underscoring that nodule size and developmental context modulate the intensity and outcome of microbial competition [162].

Further illustrating this principle, Crosbie et al. [34] identified antagonistic interactions between an intracellular *Pseudomonas* strain and an ineffective *Rhizobium* species. The *Pseudomonas* spp. isolate co-colonized nodules infected by a beneficial *Mesorhizobium* spp. but was absent from nodules formed by a non-fixing *Rhizobium* sp., implying selective interference. Moreover, another *Pseudomonas* spp. strain reduced the number of ineffective nodules induced on *Lotus japonicus* (Regel) K. Larsen by *Rhizobium* sp. BW8-2, thereby inhibiting early colonization. Intriguingly, this antagonistic effect was host-specific—observed in *Lotus japonicus* (Regel) K. Larsen but absent in *Lotus burttii Borsos* demonstrating that plant genotype modulates both the magnitude and direction of microbial competition within nodules [34]. Competitive interference extends beyond bacterial antagonists.

Interactions between AMF and rhizobia can also exhibit inhibitory cross-effects. Prior inoculation with either symbiont may constrain subsequent colonization by the other, suggesting a context-dependent rivalry for host-derived resources or signaling channels [130]. The nature and intensity of competition between rhizobia and other nodule-associated microbes depend on multiple ecological and physiological factors, including (i) whether different *Rhizobium* strains or species cohabit the same nodule or occupy distinct nodules on the same plant, and (ii) the physiological state of the rhizobia—whether free-living in intercellular spaces or differentiated into N-fixing bacteroids within plant cells [163,164].

Nodules also serve as hotspots of horizontal gene transfer, with elevated rates of plasmid conjugation facilitating the dissemination of competitive traits among co-infecting strains [18]. This genetic fluidity likely enhances adaptive potential and cross-protection in shared or successive symbiotic environments. Nodule occupancy, a key integrative metric of symbiotic fitness, reflects the cumulative success of microbial partners through successive developmental stages—rhizosphere colonization, infection thread proliferation, and persistence within the nodule [165]. However, current methods for assessing nodule occupancy do not resolve which stage exerts the greatest selective pressure [18]. Collectively, these findings reveal that competition within nodule microbiomes is multifaceted and dynamic, encompassing spatial exclusion, metabolic interference, and genetic exchange. Such complexity challenges the traditional view of nodules as exclusive sanctuaries for cooperative symbionts. Instead, nodules emerge as microbial battlegrounds, where shifting rivalries sculpt community composition, nitrogen fixation efficiency, and plant fitness. Deciphering these hidden conflicts—through the integration of spatial ecology, multi-omics, and real-time imaging—will be central to understanding and harnessing the evolutionary ecology of symbiosis for sustainable agriculture.

#### 3.2.2. Chemical Arsenal of the Microbial Arena: Secreting Strategies in Nodule Microbiomes

Beyond cooperative nutrient exchange, root nodules also function as biochemical arenas where microbial residents deploy an arsenal of secreted molecules to secure competitive advantage and spatial dominance. Nodule-inhabiting bacteria actively produce antimicrobial compounds, including gramicidins and cyclic tyrocidine peptides, which can shape both the structure and the function of the resident microbiome [46]. The biosynthesis of these compounds not only mediates antagonistic interactions among community members but may also protect the nodule from external pathogens.

Theoretical frameworks of microbial ecology predict that toxin production is favored under conditions of moderate spatial mixing, where competing lineages interact locally and resource limitation triggers strong selection for interference competition [161]. In these contexts, microorganisms engage in both chemical and contact-dependent attacks, eliminating competitors through antibiotic secretion or the direct injection of toxic effectors. For example, *Pseudomonas aeruginosa* launches Type VI Secretion System (T6SS)-mediated assaults in response to bacterial antagonism, whereas *Vibrio cholerae* and *Pseudomonas fluorescens* secrete extracellular matrix polymers that confer a positional advantage—physically excluding competitors from nutrient-rich zones. This strategy is a bacterial “nano-weapon” used to eliminate rival bacteria within the limited, nutrient-rich environment of root nodules. These strategies exemplify the highly localized and resource-driven nature of microbial conflict within nodules.

A wide array of adaptive traits underlies microbial competitiveness for nodule occupancy, encompassing:

(i) chemotactic responsiveness to seed and root exudates [166];
(ii) effective communication with host signaling networks [167];
(iii) metabolic versatility in catabolizing diverse carbon sources [168];
(iv) the synthesis of bacteriocins and other narrow-spectrum toxins targeting rhizobial competitors [169]; and
(v) tolerance to oxidative, osmotic, and nutrient stresses characteristic of the nodule microenvironment [170].

Host factors further modulate these dynamics: legume-encoded incompatibility determinants can dictate strain-specific compatibility and exclusion, thereby influencing the outcome of microbial contests during both nodule formation and maintenance [171]. Consequently, competition for nodule occupancy reflects a complex interplay among rhizobial genotype, host genotype, and community context, forming a tripartite axis of ecological selection [18].

Beyond classical antibiotics, some rhizobia produce specialized metabolites that mediate both competition and cooperation. A prominent example is the class of rhizopines—inositol-derived signaling compounds produced exclusively within nodules by specific *Rhizobium* spp. and *Sinorhizobium* spp. strains [172]. The canonical rhizopines scyllo-inosamine (SI) and 3-O-methyl scyllo-inosamine (3-O-MSI) are synthesized via a two-step pathway involving an ononitol dehydrogenase (mosDEF) and an aminotransferase (mosB), both regulated by the nitrogenase master regulator NifA [8]. Bacteria capable of producing rhizopines typically possess the mocRABCDEF operon, enabling rhizopine catabolism [172,173].

It is hypothesized that rhizopine-catabolizing strains gain a fitness advantage within nodules by utilizing rhizopines as exclusive carbon or nitrogen sources. This advantage appears most pronounced during early symbiotic stages, though whether it arises from rhizopine catabolism or the metabolism of related plant- or microbe-derived compounds remains uncertain [172]. Interestingly, genes for rhizopine utilization have also been detected in non-rhizobial taxa [172,174], implying that rhizopines may act as a shared metabolic currency or “public good” within the nodule community—accessible to all microbes equipped for its degradation.

This dual nature—where secreted molecules simultaneously foster competition and cooperation—illustrates the evolutionary paradox of the nodule microbiome. While antimicrobial production enforces spatial and nutritional segregation, metabolite sharing such as rhizopine exchange promotes metabolic interdependence and collective resilience. Together, these processes define the chemical ecology of the nodule: a finely balanced system of warfare and reciprocity that governs microbial coexistence, shapes nitrogen-fixing efficiency, and ultimately influences host fitness.

All the discussed intricate interactions among the nodule-associated microorganisms are regulated and orchestrated in the frame of plant immunity system to foster this inter-kingdom ecosystem functioning and sustainability.

## 4. Regulation of Nodule-Associated Microbiome: The Intricacies of Plant Immunity Regulation in Rhizobia-Legume Symbiosis

Leguminous plants harness the nitrogen-fixing capabilities of rhizobia that inhabit specialized root organs known as nodules. This remarkable mutualism, which converts atmospheric nitrogen into biologically accessible forms, depends on the activity of live, metabolically active nodule cells that, intriguingly, do not trigger robust defense responses despite being colonized by high bacterial densities. This paradox raises a central question in symbiotic biology: How is plant immunity modulated to permit rhizobial colonization while maintaining overall immune vigilance, and what role does the broader nodule-associated microbiome play in this regulatory balance?

Unlike animals, which possess specialized immune cells, plants rely on innate immunity, activated upon recognition of conserved microbial signatures known as microbe- or pathogen-associated molecular patterns (MAMPs/PAMPs) [175]. These molecules—ubiquitous across microbial taxa—are sensed by pattern-recognition receptors (PRRs) on the plant cell surface, initiating downstream defense cascades that include mitogen-activated protein kinase (MAPK) signaling and the activation of MAMP-triggered immunity (MTI) [176,177]. Given the high density of rhizobia within nodules, it is striking that MTI is not robustly activated in these tissues. Whether rhizobial PAMPs can elicit classical immune responses in their host remains unresolved, although structural components such as lipopolysaccharides (LPS) and exopolysaccharides—key constituents of rhizobial cell envelopes—are potential triggers. The mild and transient defense-like reactions observed during the early stages of infection suggest that rhizobial PAMPs are indeed perceived, but that defense signaling is subsequently suppressed to allow symbiosis establishment [178].

Thus, even though nodulation represents a mutualistic interaction, the plant’s innate immune machinery is transiently activated upon bacterial entry, paralleling early pathogen responses. However, the precise role and regulation of these initial defenses during nodulation remain poorly characterized. Rhizobia have evolved an array of signaling molecules that neutralize plant immunity, facilitating root invasion and nodule colonization [177,179]. During the initial recognition phase, legumes employ a dual-layered perception system distinguishing between pathogens and beneficial symbionts. Nod factors (lipochitooligosaccharides) and rhizobial effector proteins function as key modulators—suppressing immune activation while simultaneously triggering nodulation signaling cascades [175]. Secondary signals, notably LPS and exopolysaccharides, further refine this process by selecting compatible rhizobial partners and dampening immune responses during infection thread formation [36,177,180]. Rhizobial exopolysaccharides, in particular, appear to serve as immune suppressants that facilitate invasion and nodule organogenesis [177,181]. Aslam et al. [182] demonstrated that exopolysaccharides from *Sinorhizobium meliloti* can attenuate MTI in *Arabidopsis* by chelating cytosolic calcium—an essential second messenger in defense signaling—thereby blocking immune responses triggered by the bacterial flagellin peptide flg22. In legumes, exopolysaccharides perception is mediated by the exopolysaccharide receptor 3 (EPR3), which recognizes compatible exopolysaccharides structures and suppresses downstream defense activation, though the precise molecular mechanism of EPR3-mediated immunity modulation remains unclear [175].

Following this early immune attenuation, mature nodules exhibit near-complete suppression of defense activity, implying the establishment of a localized state of immune tolerance [177]. Mechanistically, this suppression parallels certain strategies observed in pathogenic interactions, including PAMP divergence, Ca^2+^ sequestration, effector-mediated interference, and hormonal pathway modulation [178,183]. Importantly, legumes appear to employ symbiosis-specific immune suppression mechanisms that prevent self-destruction of the nitrogen-fixing organ while maintaining surveillance against non-symbiotic microbes. The extent to which this tolerance extends to other members of the nodule-associated microbiome remains an open question of significant ecological and evolutionary importance.

**Figure 3:**
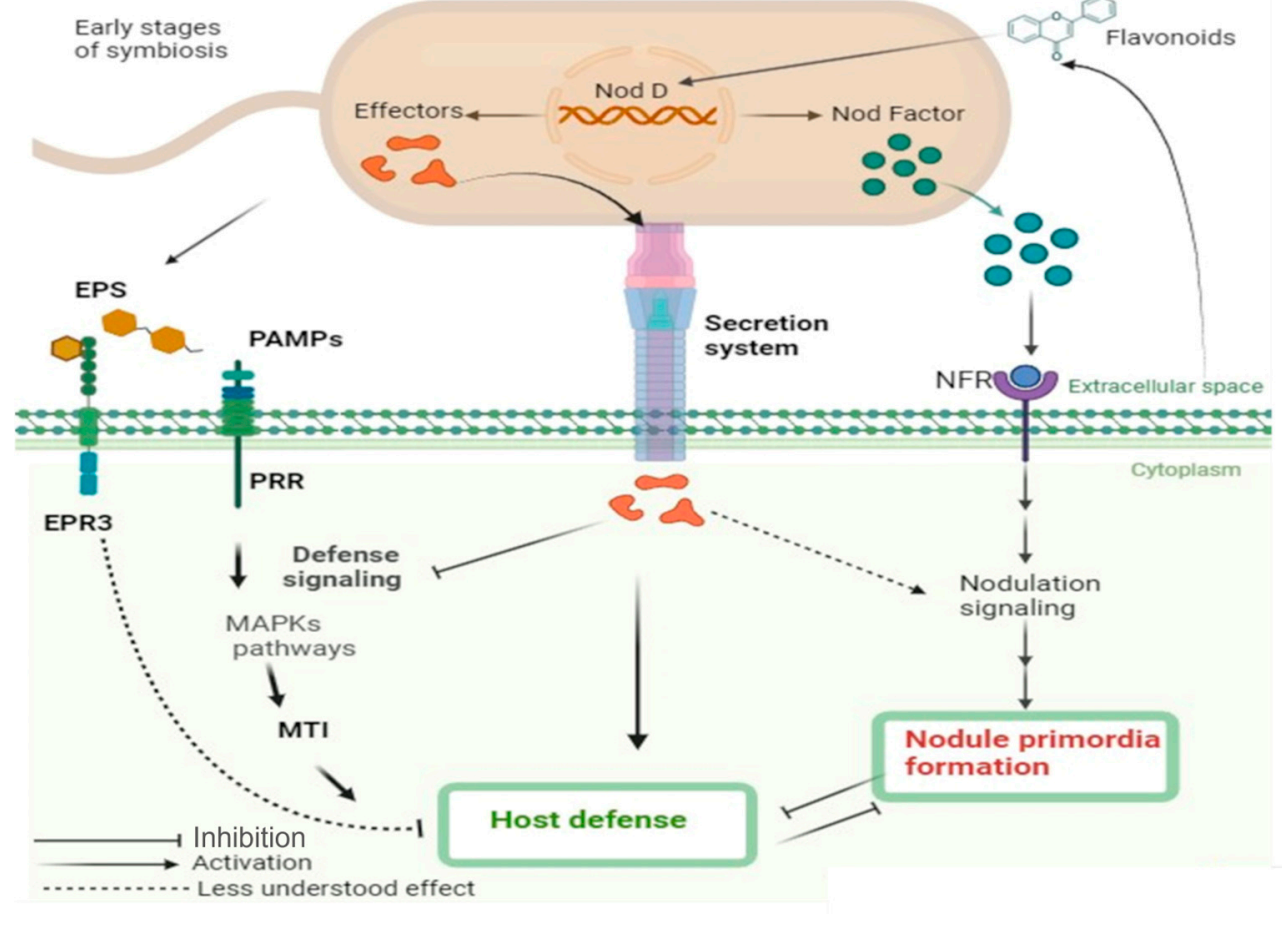
Dynamic interactions in rhizobia–legume symbiosis: orchestrating immune responses and microbial regulation. This schematic illustrates the molecular interplay between host immune signaling and rhizobial symbiotic signaling during early stages of legume nodule formation. Recognition of microbe- or pathogen-associated molecular patterns (*MAMPs*/*PAMPs*) by pattern-recognition receptors (*PRRs*) activates defense cascades via mitogen-activated protein kinases (*MAPKs*), culminating in MAMP-triggered immunity (*MTI*) and transient defense responses. Concurrently, rhizobial secretion systems release Nod factors, which are perceived by Nod factor receptors (NFRs) to initiate nodulation signaling and nodule primordia formation. Rhizobial exopolysaccharides interact with EPR3 to modulate host immunity, attenuating *MTI* and enabling bacterial infection thread progression. Other symbiotic signals, including flavonoids and secondary effector molecules, fine-tune this signaling balance by suppressing excessive defense activation and promoting compatible rhizobial colonization. Collectively, these processes establish a delicate equilibrium between host defense suppression and symbiotic accommodation, ensuring successful nodule initiation and the formation of a functional nitrogen-fixing organ.

Molecular genetics has provided key insights into host genes that govern this immune modulation during symbiosis. In *Medicago truncatula* Gaertn, several genes have been identified that sequentially restrict defense activation in nodules, including *DNF2*, *SymCRK*, *RSD*, and *NAD1* [178,184,185]. *SymCRK* encodes a receptor-like kinase expressed in rhizobia-containing cells and carries a non-arginine-aspartate kinase motif typical of PRRs, suggesting a possible role in interference with immune coreceptors [178]. Mutants in *dnf2* and *symCRK* exhibit ethylene-dependent defense phenotypes, implicating hormonal signaling in the modulation of symbiotic immunity [186]. *RSD*, encoding a transcriptional regulator, has been proposed to suppress vesicle trafficking by downregulating *VAMP721A* expression, thereby influencing symbiosome development [187]. *NAD1* likely acts later in the infection process, though its precise temporal role remains undefined [178]. Genetic and phenotypic analyses suggest that *RSD* and *SymCRK* act in sequence downstream of *DNF2*, orchestrating the progressive suppression of immune signaling as nodulation advances [185] (Figure 3).

Additional regulators have been uncovered in other model legumes. In *Lotus japonicus*, the gene *APN1*, encoding an aspartate peptidase with a signal peptide, functions to suppress defense responses following bacterial internalization in a strain-specific manner, possibly through targeting to the symbiosome [188]. However, the molecular mechanisms linking *APN1* to immune tolerance remain to be elucidated.

Recent experiments underscore the sensitivity of nodulation to perturbations in plant immune homeostasis. *Medicago Truncatula* Gaertn co-inoculated with the mutualist *Sinorhizobium medicae* and the pathogen *Ralstonia solanacearum* exhibited severe inhibition of nodulation, an effect dependent on the Type III secretion system (T3SS) of the pathogen [189]. These finding highlights how immune pathways regulating pathogen-triggered immunity (PTI) can also constrain symbiotic infection, suggesting potential crosstalk between beneficial and pathogenic signaling networks.

Despite these advances, the molecular circuitry orchestrating immune tolerance in nodules remains incompletely understood. The identification of plant genes, receptors, and signaling nodes mediating the delicate balance between immunity and symbiosis is an urgent frontier in plant–microbe research. A deeper understanding of these processes will not only shed light on the evolution of host–microbe coexistence but also guide the development of elite rhizobial inoculants and legume cultivars optimized for robust symbiosis under field conditions.

## 5. Harnessing Root Nodule Bacteria for Plant Growth

### 5.1. Navigating the Symbiotic Nexus: Unraveling Nodule Traits That Power the Host Plant

Legume–rhizobium symbiosis represents one of nature’s most sophisticated examples of inter-kingdom cooperation, where specialized organs—the root nodules—serve as microaerobic sanctuaries for BNF. Two genes, *NOOT* and *COCH*, have recently been identified as critical regulators of nodule development and maintenance, functioning to repress root identity and stabilize the symbiotic organ’s unique differentiation program [5,190]. Nodules are broadly classified as determinate or indeterminate, depending on meristem persistence, with each type exhibiting distinct developmental dynamics and metabolic zonation [191].

In both systems, rhizobia convert atmospheric nitrogen into ammonium, providing the host with a readily assimilable N source, while the plant reciprocally supplies carbon-rich photosynthates that sustain rhizobial metabolism and reproduction [192,193]. Despite the centrality of this metabolic reciprocity, the precise molecular and cellular mechanisms coordinating carbon–nitrogen exchange remain incompletely understood.

Recent advances in single-cell and spatial transcriptomics have begun to unravel the cell-type-specific architecture of nodules. Using soybean as a model, Sun et al. [13] delineated the metabolic compartmentalization of the ureide biosynthetic pathway, revealing how individual cell types coordinate nitrogen assimilation during active fixation. RNA velocity analyses further indicated that uninfected nodule cells originate from outer cortical tissues, whereas the inner cortex derives from pericycle-bound procambial cells, highlighting distinct lineage trajectories within the organ. Functional analyses of two transcriptional regulators, *GmbHLH93* and *GmSCL1*, confirmed their roles in nodulation, while enrichment of cytokinin-responsive genes identified GmCRE1, a cytokinin receptor, as a pivotal regulator of nodule differentiation. Inactivation of *GmCRE1* resulted in severe nodulation defects characterized by a diminished N-fixing zone, depletion of leghemoglobins, and widespread downregulation of nodule-specific genes [13].

Cytokinin signaling plays a central role in nodule organogenesis, activating cortical cell divisions that underlie pseudo-nodule formation in legumes. Evolutionary reconstructions suggest that cytokinin pathways were integrated into the Common Symbiosis Signaling Pathway (CSSP) through the recruitment of the transcription factor *Nodule Inception* (*NIN*), whose promoter acquired novel regulatory regions conferring responsiveness to cytokinin [13,194,195]. This innovation likely enabled early legumes to evolve nodule primordia via cortical reprogramming—a key step in the emergence of nitrogen-fixing symbiosis.

Enhancing symbiotic efficiency remains a major goal in sustainable agriculture. Strategies include boosting rhizobial density in the rhizosphere, improving nitrogenase activity, and delaying nodule senescence. Zhou et al. [191] comprehensively characterized the structural, physiological, and genetic dimensions of nodule senescence, emphasizing the coordinated roles of cysteine proteases, transcription factors, cystatins, Nodule Cysteine-Rich (NCR) peptides, and hormone- and stress-responsive genes [191,196]. These studies provide molecular frameworks for selecting varieties with delayed senescence, thereby prolonging N fixation activity. However, the regulatory hierarchy among cysteine proteases and their interplay with hormonal and environmental cues remains poorly understood.

Among signaling molecules, NO has emerged as a key regulator of nodule aging and turnover [191,197]. Acting as both a signal and a potential cytotoxin, NO contributes to the orchestration of nodule senescence, yet its downstream targets remain elusive. Plant hormones—abscisic acid, ethylene, gibberellins, and jasmonic acid—also influence senescence, although the cross-regulatory mechanisms among these pathways are only beginning to be resolved [198]. The next frontier lies in elucidating how NO–hormone crosstalk and environmental stress integration jointly regulate the onset and progression of nodule senescence.

Beyond nitrogen fixation, rhizobia contribute to plant defense and resilience by inducing systemic resistance against pathogens. *Medicago truncatula* Gaertn and *Pisum sativum* L. inoculated with rhizobia exhibit enhanced resistance to fungal pathogens, associated with elevated salicylic acid (SA) levels and the induction of SA-dependent defense markers [199,200]. This suggests that rhizobia can function as biocontrol agents, priming host immunity through noncanonical signaling routes.

Legume nodules also host a diverse non-rhizobial microbiome, which complements rhizobia by contributing to stress tolerance, nutrient cycling, and disease suppression [35]. Many of these bacteria synthesize plant growth-promoting substances such as IAA, ACC deaminase, phosphatases, chitinases, siderophores, and lytic enzymes, while others release VCs that bolster plant defense and stress adaptation [41,47,99,103].

Some members of the nodule microbiome, such as *Pseudomonas putida*, harbor mobile genetic elements encoding metal resistance genes (Cd, Ni, Zn, Co), conferring tolerance to contaminated soils [201,202]. Given the frequent cohabitation of *Pseudomonas* species within nodules [34], such traits may be horizontally transferred to rhizobia, broadening their environmental resilience. Similarly, stress tolerance genes enabling survival under high temperature and pressure have been detected on genomic islands in β- and γ-proteobacteria [202,203]. Remarkably, recent evidence suggests that horizontal gene transfer (*HGT*) can occur not only among microbes but also between plants and their associated microbiota. In *Arabidopsis*, genomic analyses revealed multiple *HGT* events involving abiotic stress-resistance genes of microbial origin, implying that such exchanges may have shaped plant adaptation to environmental pressures [202]. These discoveries underscore a deep coevolutionary continuum between legumes and their nodule microbiomes, where gene flow, signaling plasticity, and metabolic integration collectively enhance plant performance under diverse ecological conditions.

### 5.2. Orchestrating Plant Growth Through Targeted Nodule Microbiome Applications

The nodule-associated microbiome plays a multifaceted role in plant growth, nutrient assimilation, and stress adaptation, acting as a biochemical and ecological interface between plants and soil. Microbes inhabiting root nodules possess the capacity to mobilize essential nutrients, enhance soil physicochemical properties, and modulate key signaling compounds including phytohormones, secondary metabolites, and antimicrobial agents [41,47,99,103]. Collectively, these processes contribute to enhanced plant vigor, resilience, and productivity under both optimal and stressful conditions.

Microbial strains isolated from the root nodules of *Mimosa pudica* L.—notably *Enterobacter* and *Serratia* species—have demonstrated broad-spectrum PGP traits such as P solubilization, auxin production, and cellulase and chitinase activity. Remarkably, these isolates successfully colonized heterologous hosts such as *Phaseolus vulgaris* L., promoting both vegetative and reproductive growth [141]. Similarly, *Amphicarpaea bracteata* (L.) Fernald-derived nodule PGPR strains have been shown to confer stress tolerance and yield enhancement in soybean through multifactorial effects on nutrient metabolism and hormonal balance [204].

The nodule microbiome also contributes indirectly to nitrogen economy by reducing dependence on synthetic fertilizers—either by supporting rhizobial N fixation or by secreting metabolites that enhance the activity of N-fixing symbionts [205]. Nitrogen-fixing microbes are broadly categorized into symbiotic and free-living fixers. Symbiotic groups—including *Allorhizobium* spp., *Azoarcus* spp., *Azorhizobium* spp., *Bradyrhizobium* spp., *Burkholderia* spp., *Frankia* spp., *Mesorhizobium* spp., *Rhizobium* spp., and *Sinorhizobium* spp.—form intimate associations with plant roots, while free-living N fixers such as *Azospirillum* spp., *Azotobacter* spp., *Gluconacetobacter* spp., and *Herbaspirillum* spp. independently enrich soil nitrogen pools [206,207]. Within this symbiotic continuum, vascular rhizobial endophytes represent a particularly intriguing category. The model strain *Azorhizobium caulinodans*, *ORS571*, a nodule endophyte of *Sesbania rostrata*, produces cellulases and pectinases that facilitate colonization of xylem elements during nodule establishment [208,209]. This ability to inhabit vascular tissues underscores the evolutionary versatility of rhizobia and highlights the metabolite exchange occurring between the symbiont and host via xylem-mediated transport [210].

While the development of microbial inoculants often prioritizes strains showing optimal performance under controlled conditions, their nodule occupancy rates and ecological competitiveness in natural soils remain critical [200,211,212,213]. Native soil communities often outcompete commercial inoculants, particularly in smaller nodules where niche space is limited [163]. Moreover, inoculation benefits may persist in soils for up to 90 days, but long-term effects depend on environmental stability and microbial adaptability) [214].

Host-specific interactions further shape these outcomes. For instance, in *Lotus japonicus* (Regel) K. Larsen, *Pseudomonas* spp. preferentially colonize efficient nodules, while suppressing the formation of ineffective ones, suggesting that specific nodule-associated microbes may act as symbiotic quality controllers [34]. These findings point toward the potential of synthetic microbial consortia designed to emulate beneficial community structures and interactions observed in nature—enabling precision microbiome engineering for legume crops in degraded or nutrient-poor soils.

The application potential of nodule microbiomes extends beyond legumes. In lentil (*Lens culinaris* Medik), inoculation with nodule-derived bacteria *Serratia plymuthica* 33GS and *Serratia* sp. R6 significantly enhanced plant growth and root hair formation while reshaping rhizospheric community structure [36]. Scanning electron microscopy confirmed successful colonization, while metabolomic profiling revealed alterations in root exudate composition, suggesting that microbial activity modulates root metabolite secretion patterns [215,216].

Such modulation mirrors the SIREN (Systemically Induced Root Exudation of Metabolites) phenomenon described in tomato, where *Bacillus subtilis* triggers the release of targeted root metabolites that restructure rhizospheric microbial communities [217]. In lentil, inoculation with *Serratia plymuthica* 33GS or *Serratia* sp. R6 stimulated the exudation of triterpenes (e.g., 4,6-cholestadien-3β-ol and stigmast-5-en-3β-ol), fatty acids, and methyl esters, which collectively fostered the proliferation of beneficial taxa such as *Rhizobium* spp., *Mesorhizobium* spp., and *Bradyrhizobium* spp. [36].

Correlation network analyses further revealed cooperative relationships among bacterial taxa, suggesting that metabolite-mediated communication underpins microbe–microbe synergies within the rhizosphere. Many of these compounds—especially triterpenes—are known to modulate microbial colonization and enhance host immunity [218,219]. Fatty acids and isoprenoids, including pentadecane (2,6,10,14-tetramethyl-1-(methylsulfonyl)), have also been implicated in plant development and pathogen suppression [36,220,221,222].

Taken together, these studies reveal that targeted manipulation of nodule microbiomes—through the selection, inoculation, or engineering of beneficial strains—can extend beyond N fixation to orchestrate systemic effects on plant physiology and rhizosphere ecology. This emerging paradigm transforms the nodule from a simple symbiotic organ into a metabolic and signaling hub, capable of shaping plant performance through multi-kingdom biochemical communication.

### 5.3. Paving the Way for Next-Generation Agriculture with Nodule-Associated Microbiomes

Most contemporary strategies seeking to harness microbial diversity for crop improvement have focused on developing elite microbial inoculants, typically composed of a few highly effective strains selected under field conditions. These inoculants—common in legume-based systems across many countries—are formulated through the isolation, screening, and performance evaluation of strains demonstrating superior competitiveness, colonization ability, and plant-growth-promoting capacity. However, while this approach has delivered incremental benefits, it overlooks the vast functional potential embedded within the nodule microbiome, whose diversity remains largely untapped [193].

Beyond their direct contribution to plant nutrition and health, nodule-associated microbes represent a reservoir of adaptive genetic and metabolic traits that could be leveraged for sustainable agriculture. Historically, plant breeding has emphasized the manipulation of host genetic variation to develop high-yielding, disease-resistant, or drought-tolerant cultivars. Yet, the growing realization that plant performance is co-determined by its associated microbiome opens new horizons for “microbiome-assisted breeding”—the design of crops that not only exploit their own genetic potential but also recruit, sustain, and cooperate with beneficial microbial partners. This paradigm holds promise for the development of next-generation crops that require fewer chemical inputs, maintain resilience under climatic stress, and exhibit enhanced protection against pathogens and pests.

Synthetic biology offers transformative opportunities to accelerate this transition. By reprogramming microbial physiology and host–microbe communication, synthetic biology can be used to optimize microbial functions that directly impact plant growth. At the microbial level, targeted manipulation of gene networks may enhance nitrogen fixation, phosphorus solubilization, or phytohormone biosynthesis. At the host level, synthetic circuits can be engineered to fine-tune gene expression, improve signal perception, or reshape root exudate chemistry to favor beneficial microbial recruitment [223].

**Figure 4:**
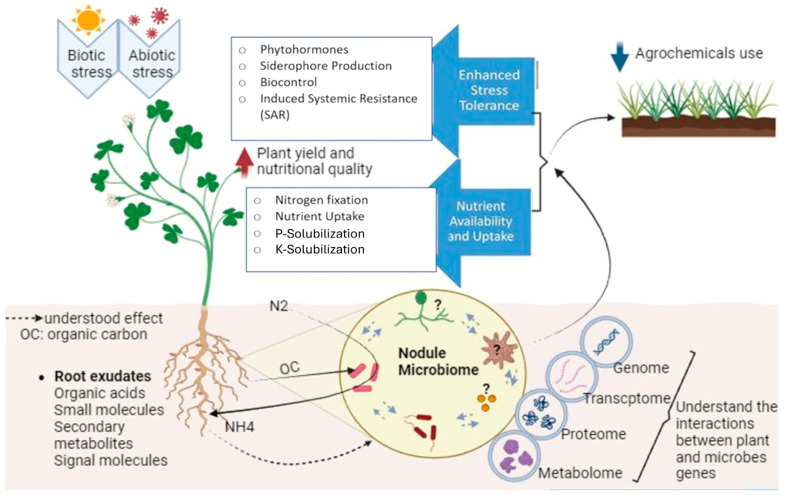
Dynamic interplay within the root nodule microbiome during symbiotic interaction. This conceptual model illustrates how the nodule microbiome—comprising rhizobia, non-rhizobial bacteria, fungi, and protists—acts as a central hub for nutrient exchange, stress resilience, and metabolic signaling within the legume root system. Through coordinated processes such as BNF, nutrient solubilization (P and K), and organic carbon cycling, the microbiome enhances both plant yield and nutritional quality. Microbial secretion of phytohormones, siderophores, and secondary metabolites promotes induced systemic resistance (ISR) and tolerance to biotic and abiotic stressors, reducing dependence on agrochemicals. Arrows denote bidirectional interactions between host roots and microbial communities mediated by root exudates (organic acids, amino acids, secondary metabolites, and signaling molecules). The lower right panel highlights multi-omics approaches—genome, transcriptome, proteome, and metabolome analyses—as essential tools for elucidating gene-level interactions between plants and their associated microbes. Collectively, these processes underscore the potential of nodule-associated microbiomes as living bio-platforms for sustainable, climate-smart agriculture.

Emerging molecular technologies such as CRISPR/Cas-based editing (Cas9, Cas12a), gene silencing, and overexpression systems enable precise reconfiguration of host or microbial genomes, while advanced omics approaches—including amplicon sequencing, integrative metagenomics, metabolomics, and metatranscriptomics—offer unprecedented insights into nodule microbiome dynamics [28]. These complementary tools bridge host-centered and microbe-centered disciplines, enabling bidirectional engineering of symbiosis for optimized performance (Figure 4).

Despite these advances, the application of synthetic biology to nodule microbiomes remains in its infancy. Only a handful of studies have explored how engineered microbial consortia might enhance nitrogen fixation, improve nutrient uptake (e.g., phosphorus), or modulate plant hormone homeostasis in situ [224,225]. The next frontier lies in synthetic design of functional nodule ecosystems—communities whose members are tailored not only for biochemical competence but also for ecological stability, genetic compatibility, and cooperative performance within the host.

Harnessing this potential marks a paradigm shift from descriptive to design-driven microbiome science. By integrating systems biology, genetics, and synthetic design, nodule-associated microbiomes could become living biofactories—sustainably powering plant productivity, ecosystem resilience, and carbon–nitrogen balance in the era of climate-smart agriculture.

## 6. Conclusions

This review synthesizes current advances in our understanding of the root nodule microbiome, a complex and dynamic consortium of microorganisms that extends far beyond the classical rhizobium–legume paradigm. By exploring the diversity, interactions, and functional potential of nodule-associated bacteria, archaea, fungi, and protists, we highlight the intricate ecological and molecular processes that underpin nodule formation, maintenance, and their contribution to plant growth. Together, these findings redefine the root nodule as a multispecies symbiotic organ, where metabolic cooperation, signaling exchange, and competitive dynamics converge to sustain plant productivity and resilience.

Mounting evidence confirms that the nodule microbiota is integral to plant health, nutrient acquisition, and yield improvement. Yet, this subterranean ecosystem remains only partially understood. A deeper characterization of its structure, function, and inter-kingdom communication is imperative to elucidate how microbial networks coordinate colonization, nutrient exchange, and defense modulation within this specialized niche. In particular, greater attention should be given to the fungal and protist components of the nodule microbiome, whose ecological and physiological roles are emerging as critical yet underexplored dimensions of symbiosis.

Future research should adopt systems-level and molecular approaches to decode the genetic, metabolic, and regulatory circuits that govern nodule microbiome assembly and function. Cutting-edge tools—ranging from multi-omics integration to nanoscale imaging, CRISPR-based functional genomics, and synthetic biology—offer unprecedented opportunities to map microbe–microbe and plant–microbe interactions with high spatial and temporal resolution. Such methodological innovation will enable the transition from correlative to causal understanding of nodule-associated microbiota.

In an era marked by climate volatility, soil degradation, and population pressure, unraveling the full potential of nodule microbiomes stands as a promising frontier for sustainable and climate-smart agriculture. By transforming our understanding of nodules from nitrogen-fixing organs into dynamic bioengineered ecosystems, we can pave the way for microbial solutions that enhance productivity, reduce agrochemical dependency, and restore ecological balance. The future of legume science—and perhaps of agriculture itself—will depend on how effectively we harness this hidden microbial world to sustain life above and below ground.

## Data Availability

No new data were created or analyzed in this study.
